# Clinical and Functional Features of Epilepsy-Associated In-Frame Deletion Variants in *SCN1A*

**DOI:** 10.3389/fnmol.2022.828846

**Published:** 2022-03-14

**Authors:** Jing-Yang Wang, Bin Tang, Wen-Xiang Sheng, Li-Dong Hua, Yang Zeng, Cui-Xia Fan, Wei-Yi Deng, Mei-Mei Gao, Wei-Wen Zhu, Na He, Tao Su

**Affiliations:** ^1^Institute of Neuroscience, The Second Affiliated Hospital of Guangzhou Medical University, Guangzhou, China; ^2^Key Laboratory of Neurogenetics and Channelopathies, Ministry of Education of China, Guangzhou, China; ^3^Department of Neurology, The Second Affiliated Hospital of Guangzhou Medical University, Guangzhou, China; ^4^Translational Medicine Center, Maternal and Child Health Research Institute, Guangdong Women and Children’s Hospital, Guangzhou, China

**Keywords:** sodium channel, *SCN1A*, epilepsy, in-frame deletion, variant

## Abstract

**Objective:**

Naturally occurring in-frame deletion is a unique type of genetic variations, causing the loss of one or more amino acids of proteins. A number of in-frame deletion variants in an epilepsy-associated gene *SCN1A*, encoding voltage gated sodium channel alpha unit 1.1 (Na_v_1.1), have been reported in public database. In contrast to the missense and truncation variants, the in-frame deletions in *SCN1A* remains largely uncharacterized.

**Methods:**

We summarized the basic information of forty-four *SCN1A* in-frame deletion variants and performed further analysis on six variants identified in our cases with epilepsy. Mutants of the six in-frame deletions and one truncating variant used as comparison were generated and co-transfected with beta-1 and -2 subunits in tsA201 cells, followed by patch clamp recordings.

**Results:**

Reviewing all the in-frame deletions showed that they spread over the entire Na_v_1.1 protein, without obvious “hot spots.” The dominant type (54%) was single residue loss. There was no obvious relationship between the length or locations of deletions and their clinical phenotypes. The six in-frame deletions were two single residue deletions (p.M400del and p.I1772del), one microdeletion (p.S128_F130del) and three macrodeletions (p.T303_R322del, p.T160_Y202del, and p.V1335_V1428del). They scatter and affect different functional domains, including transmembrane helices, pore region, and P-loop. Electrophysiological recordings revealed no measurable sodium current in all of the six mutants. In contrast, the truncating mutant p.M1619Ifs*7 that loses a long stretch of peptides retains partial function.

**Significance:**

The complete loss-of-function in these shortened, abnormal mutants indicates that Na_v_1.1 protein is a highly accurate structure, and many of the residues have no redundancy to ion conductance. In-frame deletions caused particularly deleterious effect on protein function possibly due to the disruption of ordered residues.

## Introduction

Voltage-gated sodium channels (Na_v_) are responsible for the generation and propagation of action potentials in excitable membrane. These channels are complexes of one α subunit in association with two auxiliary β subunits. In humans, there are nine functional α subunits (Na_v_1.1-Na_v_1.9) encoded by the genes *SCN1A*-SCN11A, with different patterns of tissue expression and biophysical properties. The α subunit of ∼2000 amino acid (AA) residues is organized in four homologous domains but non-identical domains (DI-DIV), each of which contains six transmembrane segments (S1–S6) and an additional membrane re-entrant pore loop (P-loop). Four transmembrane domains (DI–DIV) are connected by intracellular loop structures (Catterall, 2000; Goldin et al., 2000; Eijkelkamp et al., 2012).

Variants in the gene *SCN1A* encoding Na_v_1.1 α subunit have been associated with a spectrum of epilepsy disorders ranging from the relatively benign generalized epilepsy with febrile seizures plus (GEFS+) to the devastating disorder, severe myoclonic epilepsy of infancy (SMEI) (Escayg et al., 2000, 2001; Marini et al., 2009). To date, more than 1,800 epilepsy associated variants annotated for *SCN1A* have been reported in different databases, such as SCN1A database^1^, the Human Gene Mutation Database (HGMD), and the ClinVar database of NCBI. Most of these are missense variants that lead to a single amino acid substitution, while there are also a significant number of in-frame deletions and premature truncations that lacks one or more amino acids. Previous studies focused on characterizing the biophysical properties of missense and truncating variants (Lossin et al., 2003; Yamakawa, 2006; Meng et al., 2015). The in-frame deletions occurred in *SCN1A* have not been well characterized, except that an in-frame deletion (p.F1289del), located at DIII S3, was reported with no measurable sodium current (Ohmori et al., 2006). The naturally occurring in-frame deletions would be unique and useful models to explore the underlying biology of Na_v_1.1, and the genotype-phenotype relationship as well.

Here we first collected a total of 44 in-frame deletion variants in *SCN1A* from the HGMD and SCN1A database and characterized their features of clinical phenotypes and locations. To gain insights into sub-molecular gating network of Na_v_1.1, six in-frame deletions identified in our laboratory were further subject to site-directed mutagenesis experiments to determine the functional features of the shortened Na_v_1.1.

## Materials and Methods

### Public Data Collection

All available in-frame deletion variants in *SCN1A* (a total of 44 variants) were retrieved from the SNP database of the NCBI^2^, the SCN1A database (see text footnote 1) and the HGMD. The NCBI database was queried with amino acid sequences of human Na_v_1.1 to obtain the corresponding information of DNA locus, and related functional regions.

### Genetic Testing

Diagnose and treatments of the patients were conducted in our Epilepsy Center (Guangzhou, China). Clinical data including medical records, standardized questionnaires, and EEG recordings were available. The probands were assessed using a standardized protocol after providing written informed consent. This study was approved by the Research Ethics Board of the Hospital. Genomic DNAs were prepared from ethylenediaminetetraacetic acid (EDTA)-treated whole blood samples. *SCN1A* were screened for genetic abnormalities. Primers were designed to amplify all exons and the flanking intronic splice sites of the gene. The purified PCR products of polymerase chain reaction were directly sequenced. The variant was verified by a second targeted PCR and sequencing. A total of six in-frame deletion variants in *SCN1A* were identified in our genetic testing, among which three were novel and three variants (c.383 + 1A > G/p.S128_F130del, c.602 + 1G > A/p.T160_Y202del, and c.1200_1202delGAT/p.M400del) were previously reported (Depienne et al., 2009; Selmer et al., 2009).

### Mutagenesis and Heterologous Expression

To reconstitute the native brain sodium channel complex, *SCN1A* was co-expressed heterologously with human accessory β1 and β2 subunits in human tsA201 cells. The expression vectors of wild-type (WT) human sodium channel Na_v_1.1, pCMV-*SCN1A*-WT, pCD8-IRES-hβ1, and pGFP-IRES-hβ2 that express α, β1, and β2 subunits, were kindly donated by Professor Alfred L. George Jr. To improve the monitoring of transfection, pCD8-IRES-hβ1 had been modified into pDsred-IRES-hβ1 with red fluorescence; whereas pGFP-IRES-hβ2 expression is recognized by green fluorescence. The mutant vectors were generated from corresponding WT vectors using Quick-change site-directed mutagenesis (Stratagene, Cedar Creek, TX, United States) according to the manufacturer’s protocol. All constructs were verified by resequencing before being transfected to human tsA201 cells. The cells were grown in 1:1 Ham’s F-12 and Dulbecco’s modified eagle’s medium (DMEM) supplemented with 10% fetal bovine serum, 100 U/ml of penicillin, and 100 μg/ml streptomycin. The cells were maintained in a humidified incubator at 37°C with 5% CO_2_. Cells were then co-transfected with pCMV-*SCN1A*, pCD8-IRES-hβ1, and pGFP-IRES-hβ2, using Lipofectamine 3000 reagent Kit from Thermo Fisher Scientific. After incubation for 12–15 h, cells were replated in 35-mm culture dishes.

### Patch Clamp Analysis

Electrophysiological studies were performed 20–48 h after transfection, according to our previous report (Chen et al., 2015). Cells displaying green and red fluorescence were chosen for recording. According to our previous experience, almost all the cells (>90%) that had green and red fluorescence were expressing a complex of co-transfected α, β1 and β2 subunits. Whole-cell patch clamp was performed according to previous reports. Sodium currents were recorded from tsA201 cells at room temperature (22–24°C). Series resistance (2.0–3.0 MΩ) was compensated 85–95% to assure that the command potential was reached within microseconds and with a voltage error of <4 mV. All data were acquired at 10–50 kHz and low-pass filtered at 5 kHz. The pipette solution contained (in mM): NaF 10, CsF 110, CsCl 20, EGTA 2, and HEPES 10, with a pH of 7.35 and osmolarity of 310 mOsM/kg. The extracellular solution contained (in mM): NaCl 145, KCl 4, CaCl_2_ 1.8, MgCl_2_ 1, and HEPES 10, with a pH of 7.35 and osmolarity of 310 mOsM/kg. Sodium currents were recorded with EPC10 amplifiers (HEKA Elektronik, Lambrecht, Germany). Sodium currents were recorded at various test potentials from a holding potential of −120 mV. The inward currents were validated by Na_v_ blocker tetrodotoxin. Sodium conductance (G) was calculated according to the equation G = I_peak_/(V_test_-V_rev_), where I_peak_ is the peak inward current, V_test_ is the test potential, and V_rev_ is the reversal potential for Na^+^. To compare voltage dependence of activation, data were fitted to a Boltzmann function, according to the equation G/G_max_ = 1−1/{1 + exp[(V_m_–V_1/2_)/k]}, where G_max_ is the maximum conductance, V_m_ is the potential of individual step pulses, V_1/2_ is the average half activation potential (at which G is one-half maximal), and k is the slope factor. The voltage dependence of channel availability was assessed following a prepulse to various potentials followed by 20-ms pulse to −10 mV. The normalized current was plotted against the voltage and the inactivation curves were fit with Boltzmann functions (I/I_max_ = 1/(1 + exp[(V_m_−V_1/2_)/k]) to determine the voltage for half-maximal channel inactivation (V_1/2_) and slope factor (k). Recovery from inactivation was determined using a two-pulse protocol. The peak current from the test pulse was normalized to the peak current from a prepulse and plotted against the recovery period. Data were fit with the two exponential function, I/Imax = A_f_ [1−exp(−t/τ_f_)] + A_s_ [1−exp(−t/τ_s_)], where τ_f_ and τ_s_ denote time constants (fast and slow components, respectively).

### Structural Modeling

The structures of the WT Na_v_1.1 and fragmental peptides (about 600 AA, deletion locus was covered) of the deletion variants were modeled to predict the effect of deletion mutations on protein structure by using I-Tasser^3^. PyMOL 2.3 software was used for three-dimensional protein structure visualization and analysis.

### Statistical Analysis

All data were analyzed using a combination of Fit master v2.53 (HEKA Electronics, Lambrecht, Germany), Excel 2003 (Microsoft, Seattle, WA, United States), and OriginPro 8.0 (OriginLab, Northampton, MA, United States) software. For statistical evaluation, results are shown as means ± SEM, and differences between WT and mutant channels were assessed by Student’s *t*-test. One-way analysis of variance (ANOVA) was used to compare means among different groups. Significance was assigned at *P* < 0.05.

## Results

### Features of In-Frame Deletion Variants in *SCN1A*

**FIGURE 1:**
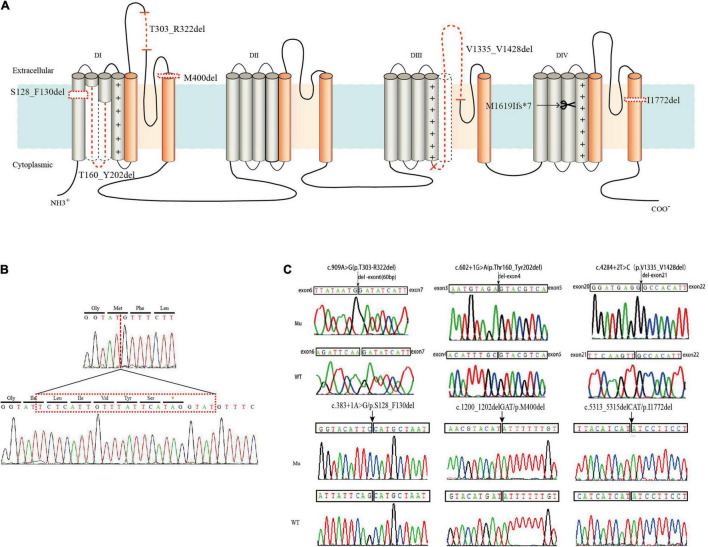
Locations of six in-frame deletion and one truncating variants in *SCN1A* and sequence identification. (A) Schematic location of the variants identified. The in-frame deletion variants were marked with dotted lines and the truncating variant was marked with a scissor. (B) DNA sequencing of the truncating variant (c.4853-25T > A/p.M1619Ifs*7). (C) DNA sequencing of the in-frame deletion variants.

Six in-frame deletions in *SCN1A* were identified in our cases with SMEI, generalized epilepsy with febrile seizures plus (GEFS+), partial epilepsy with febrile seizures plus (PEFS+) and Lennox–Gastaut syndrome (LGS) (Table 1). One variant (c.383 + 1A > G/p.S128_F130) associated with LGS and two variants (c.602 + 1G > A/p.T160_Y202del and c.1200_1202delGAT/p.M400del) associated with SMEI were reported in the ClinVar database or previous studies (Depienne et al., 2009; Selmer et al., 2009), while the other variants were novel. Residues affected by these variants are located in several distinct protein domains including the DI/S1 (c.383 + 1A > G/p.S128_F130del), DI/S2-S3 (c.602 + 1G > A/p.T160_Y202del), pore loop in DI (c.909A > G/p.T303_R322del, c.1200_1202delGAT/p.M400del), cytoplasmic DIII/S4-S5 linker to pore loops in DIII (c.4284 + 2T > C/p.V1335_V1428del), and DIV/S6 (c.5313_5315delCAT/p.I1772del) (illustrated in Figure 1).

**TABLE 1:** Clinical features associated with six identified in-frame deletion and one truncation variants in *SCN1A.*

Mutation	Mutation position	Age at FS/aFS onset	Seizure type	Inherited	Diagnosis	EEG	Mental retardation	AEDs	PROVEAN
c.383 + 1A > G/p.S128_F130del	DIS1 (Intron 2)	NA	Myo, Tonic, Atonic, CPS, GTCS	*De novo*	LGS	GSW, FSW	Severe learning disability	VPA+LTG+PT(∧)	D
c.602 + 1G > A/p.T160_Y202del	DIS2-S3 (Exon 4)	8 m/2 years	sGTCS, CPS	*De novo*	DS	FSW	Normal	VPA+TPM(↓)	D
c.909A > G/p.T303_R322del	DIS5-S6 (Exon 6)	2 years/−	GTCS	*De novo*	FS+	NA	Normal	NA	D
c.1200_1202delGAT/p.M400del	DIS6 (Exon 9)	8 m/−	GTCS, CPS	*De novo*	DS	GSW, FSW	Speech defect	VPA+LEV(↓)	D
c.4284 + 2T > C/p.V1335_V1428del	DIIIS5-S6 (Exon 21)	6 m/6 m	Myo, CPS, sGTCS	Paternal	DS	GSW, FSW	Normal	VPA+TPM(∧)	D
c.5313_5315delCAT/p.I1772del	DIVS6 (Exon 26)	7 m/3 years	GTCS, CPS	*De novo*	PEFS+	Normal	Moderate	VPA+CNZ(↓)	D
c.4853-25T > A/p.M1619Ifs*7	DIVS3 (Exon 26)	18 m/5 years	GTCS, CPS	Maternal	PEFS+	NA	NA	NA	D

*CPS, complex partial seizures; GTCS, generalized tonic-clonic seizures; Myo, myoclonic seizures; Tonic, tonic seizure; Atonic, atonic seizures; LGS, Lennox-Gastaut syndrome; GSW, generalized spike waves; FSW, focal spike waves; NA, not available; VPA, valproic acid; LTG, lamotrigine; TPM, topiramate; LEV, levetiracetam; CNZ, clonazepam; D, deleterious. (∧), seizure-free; (↓), seizure remission.*

**FIGURE 2:**
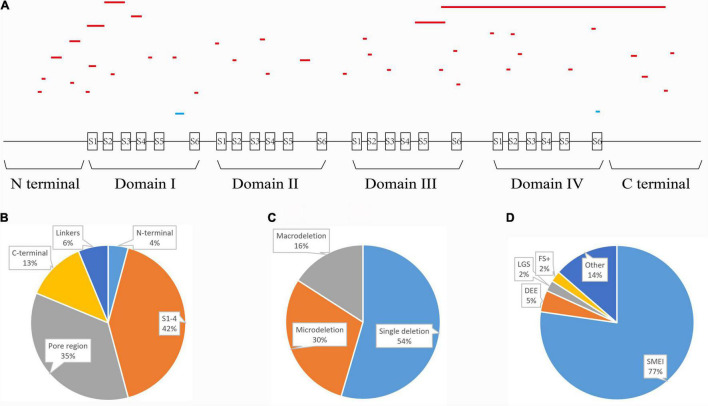
Statistics of all in-frame deletions in *SCN1A*. **(A)** Schematic picture showing the length, location, and phenotypes of the 44 variants. Lines indicate different variants. Severe phenotypes (SMEI, DEE, and LGS) were highlighted as red, while mild phenotypes (PEFS+ and FS+) were blue. **(B)** Distribution of the variants on different functional domains. **(C)** The proportions of single deletion, microdeletions, and macrodeletions. **(D)** Phenotypic spectrum.

We summarized all the in-frame deletion variants (a total of 44, together with the six variants identified by us) from the databases, and found that most of them (38/44) were reported as SMEI, developmental and epileptic encephalopathy (DEE) and LGS (Table 2). These variants spread over six functional domains of Na_v_1.1 (Figures 2A,B). Approximately 54% of the in-frame deletions are single deletions of 1 AA, followed by microdeletions (2–6 AA, 30%) and macrodeletions (>6 AA, 16%) (Figure 2C). Almost all the single deletions are associated with SMEI, DEE, except of one in DIV/S6 presented with PEFS+ Figure 2D). One microdeletion located in DI S5-S6 linker is associated with febrile seizure plus (FS+). There is no clear relationship between clinical phenotypes and the length of deletions.

**TABLE 2:** In-frame deletion variants of *SCN1A.*

Variant	Length (AA)	Position	Phenotype	References
p.Phe17del	1	N terminal	DS	Zuberi et al., 2011
p.Thr18del	1	N terminal	DS	Djemie et al., 2016
p.Gly58_Leu61del	4	N terminal	DS	https://ncbi.nlm.nih.gov/clinvar/
p.Leu80_Asp81del	2	N terminal	DS	Usluer et al., 2016
p.Ile99_Ala104del	6	N terminal	DS	Gonsales et al., 2019
p.Leu129del	1	DI S1	DS	Mancardi et al., 2006
p.Ser128_Phe130del	3	DI S1	LGS	Selmer et al., 2009
p.Leu129_Glu158del	30	DI S1, S1-S2, S2	DS	https://./clinvar/
p.Thr160_Tyr202del	43	DI S2, S2-S3, S3	DS	https://./clinvar/
p.Glu181del	1	DI S2-S3	DS	Till et al., 2020
p.Asp208_Arg219del	12	DI S3-S4, S4	DS	de Lange et al., 2018
p.Lys246del	1	DI S4-S5	DS	Peng et al., 2019
p.Leu247del	1	DI S4-S5	Ep or NDD	Lindy et al., 2018
p.Thr303_Arg322del	20	DI S5-S6	FS+	
p.Tyr325del	1	DI S5-S6	DS	Wu et al., 2015
p.Met400del	1	DI S6	DS	Depienne et al., 2009
p.Thr775del	1	DII S1	DS	Aljaafari et al., 2017
p.Met815del	1	DII S2	DEE	https://./clinvar/
p.Gly854_Leu855del	2	DII S3-S4	DS	Zuberi et al., 2011
p.Lys868del	1	DII S4	DS	Moehring et al., 2013
p.Val896_Ala898del	3	DII S5	Ep or NDD	Lindy et al., 2018
p.Met960_Cys968del	9	DII S5-S6, S6	DS	Yang et al., 2017
p.Thr1210del	1	DII-DIII linker	DS	http://SCN1A.caae.org.cn/by_mutation.php
p.Ile1240_Asp1243del	4	DIII S1-S2	DEE	https://./clinvar/
p.Thr1247_Thr1250del	4	DIII S1-S2	DS	http://SCN1A.caae.org.cn/by_mutation.php
p.Phe1289del	1	DIII S3	DS	Depienne et al., 2009
p.Val1335_V1428del	94	DIII S4-S5, S5, S5-S6	DS	
p.Ala1429del	1	DIII S5-S6	DS	Zuberi et al., 2011
p.Asn1446_Gly2008del	563	DIII S5-S6, S6, DIII-DIV, DIV, C-terminal	DS	https://./clinvar/
p.Phe1473del	1	DIII S6	DS	Depienne et al., 2009
p.Ile1483del	1	DIII S6	DS	Depienne et al., 2009
p.Glu1503del	1	DIII-DIV linker	DS	Wang et al., 2012
p.Met1558del	1	DIV S1	DS	Fukuma et al., 2004
p.Met1559del	1	DIV S1	DS	Fukuma et al., 2004
p.Val1560_Thr1562del	3	DIV S1-S2	DS	Brunklaus et al., 2020
p.Asn1672del	1	DIV S4-S5	DS	Gertler et al., 2020
p.Gly1674_Leu1675del	2	DIV S5	Ep or NDD	Lindy et al., 2018
p.Phe1766del	1	DIV S6	DS	Fukuma et al., 2004
p.Ile1772del	1	DIV S6	PEFS+	
p.Met1807_Glu1810del	4	C terminal	DS	Fujiwara et al., 2003
p.Glu1813_Phe1815del	3	C terminal	DS	Depienne et al., 2009
p.Leu1835_Pro1837del	3	C terminal	Ep or NDD	Lindy et al., 2018
p.Thr1909del	1	C terminal	DS	Zuberi et al., 2011
p.Gln1914del	1	C terminal	Refractory epilepsy	Liu et al., 2018

*DS, Dravet syndrome (SMEI); Ep, epilepsy; NDD, neurodevelopmental disorders; LGS, Lennox–Gastaut syndrome; PEFS+, partial epilepsy with febrile seizures plus; FS+, Febrile seizures plus.*

### In-Frame Deletion Mutants Exhibit Complete Loss-of-Function

**FIGURE 3:**
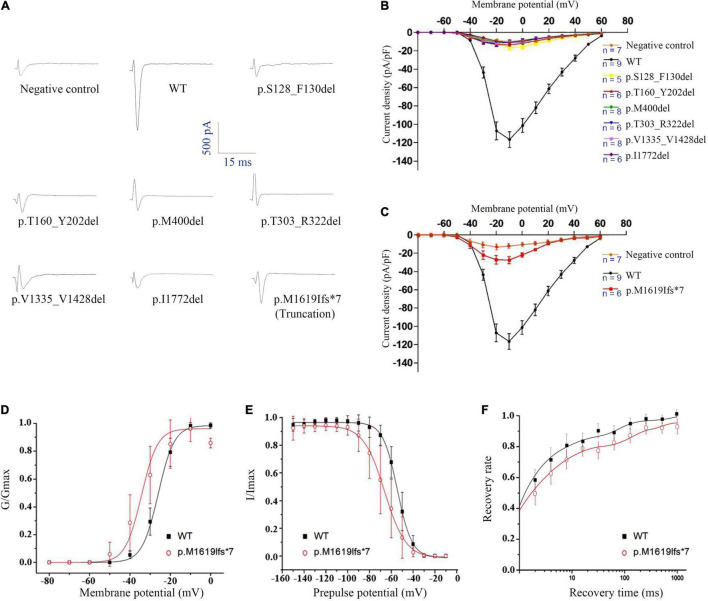
Electrophysiological analysis of the *SCN1A* in-frame deletion and truncated mutants. **(A)** Representative traces of sodium current evoked at 0 mV. **(B)** Current-voltage relationship of six in-frame deletion mutants. **(C)** Current-voltage relationship of the truncated mutant. **(D)** G-V activation curve, **(E)** steady-state inactivation curve, and **(F)** recovery from inactivation, all for the truncated mutant p.Met1619Ilefs*7. Non-transfected cells were used as negative control.

The WT and six in-frame deletion mutant alpha-subunits were expressed transiently in human tsA201 cells. We performed electrophysiological recordings for the transfected neurons and the non-transfected neurons as negative control. In contrast to the WT Na_v_1.1 with apparent current recorded (>90 pA/pF), the six in-frame deletion mutants all exhibited tiny raw currents (usually <20 pA/pF) (Figures 3A,B). The tiny currents were demonstrated as endogenous current, which was also recorded in non-transfected cells. Therefore, we confirmed that the in-frame deletion mutants were not able to generate Na^+^ current and fail to exhibit sufficient sodium current for biophysical analysis.

### A Truncated Mutant Retains Partial Channel Function

We noticed that the in-frame deletion mutant causing a single loss of isoleucine at the S6 of DIV (I1772) resulted in complete loss-of-function. This raised an interesting question about whether a truncation mutant that was much shortened still functioned. A truncating variant c.4853-25T > A/p.M1619Ifs*7 was previously identified in our patient with PEFS+. The premature truncation is expected to have seven AA substitutions from p.M1619-E1626, with a loss of peptides from S3 of DIV to c-terminal. Interestingly, the truncated mutant exhibited partial loss-of-function, with reduced peak current density (−27.47 ± 8.46 pA/pF vs. −116.60 ± 25.60 pA/pF in the WT control, 23.6% of the WT) (Figure 3C and Table 3). The current-voltage relation analysis showed that the p.M1619Ifs*7 mutant exhibited a less steep conductance-voltage (G-V) curve with the smaller slopes of steady-state availability, but remained unchanged in the half-maximal activation and inactivation (V_1/2_), suggesting a slight disruption of the voltage sensor (Figures 3D,E and Table 3). There was no significant difference in the time constants in the kinetics of recovery from inactivation (Figure 3F and Table 3).

**TABLE 3:** Biophysical parameters of truncating mutant p.Met1619Ilefs*7.

		WT (*n* = 9)	p.Met1619Ilefs*7 (*n* = 6)
Current density (−10 mv)	pA/pF	−116.60 ± 25.60	−27.47 ± 8.46^§^
Voltage-dependence of activation	V_1/2_ (mV)	−26.10 ± 4.70	−32.32 ± 25.86
	k	4.50 ± 0.15	7.31 ± 0.73^§§^
Voltage-dependence of fast inactivation	V_1/2_ (mV)	−54.42 ± 15.78	−66.03 ± 31.03
	k	6.60 ± 0.25	9.70 ± 0.42^§§^
Recovery from fast inactivation	τ_f_ (ms)	1.06 ± 0.96	1.24 ± 1.11
	τ_s_ (ms)	23.94 ± 9.43	168.86 ± 103.49

*Compared with the WT, ^§^P < 0.050, ^§§^P < 0.001.*

### Structure Modeling

**FIGURE 4:**
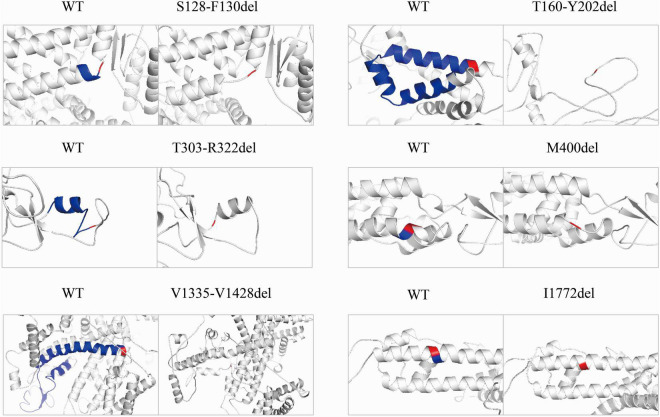
Pairwise comparison of the predicted local structure features between wild-type and in-frame deletions. Location of deleted residues is indicated in blue, and their next residues are in red.

To explore structural changes as a result of the in-frame deletions, we performed tertiary structure prediction. The comparative analysis of the predicted structures showed that the local tertiary folding differed strongly between the WT and mutants, especially for the macrodeletions (p.T303_R322del, p.T160_Y202del, and p.V1335_V1428del). The local structures of the macrodeletion mutants were deformed and the surrounding segments were relocated (Figure 4). The p.T160_Y202del deletion with two transmembrane segments involved has the most remarkable difference in protein folding, in which the expected segments (S2 and S3) disappear and moreover the adjacent S1 and S4 are relocated to the different sites. Both the single deletion (p.M400del) and microdeletion (p.S128_F130del) that have lost their hydrophobic residues including isoleucine, methionine and phenylalanine, were predicted to turn the coiled coil alpha helices into flexible loops. The other deletion (p.I1772del) at S6 exhibited a smaller difference in the alpha helix, but had a larger difference in the angle of the neighboring units compared to the WT.

## Discussion

Genetic defects in the Na_v_ α subunit can lead to various excitability diseases in brain, muscle, and heart, such as muscle paralysis, cardiac arrhythmias, and epileptic disorders (Meisler and Kearney, 2005; Lee et al., 2009; Remme and Bezzina, 2010; Kasperaviciute et al., 2013). The particular importance of Na_v_1.1 channel has continually motivated researchers to identify structural or functional residues responsible for protein stability and activity. So far, more than 1800 *SCN1A* variants have been identified in epilepsy, but few studies have investigated the function of these genetic defects. Functional analysis in coding regions of Na_v_1.1 channel might help to gain insights into the intramolecular gating network.

In this study, we report the phenotypic relevance and biophysical characterization of seven *SCN1A* variants, including six *SCN1A* in-frame deletions and one truncating variant. The six in-frame deletions scatter and affect different functional domain, including transmembrane helices, pore region, and P-loop. There are two single residue deletions (p.M400del, p.I1772del), one microdeletion (p.S128_F130del) and three macrodeletions (p.T303_R322del, p.T160_Y202del, and p.V1335_V1428del). In accordance to the features of all in-frame deletion variants summarized from public databases, the six deletion variants were associated with severe phenotypes such as SMEI, DEE, and LGS, except that two cases presented with milder phenotypes, FS+ and PEFS+. In spite of different locations, residue length of deletions, and associated phenotypes, the six in-frame deletion mutants were found to consistently lose their ion conductance, showing barely detectable inward sodium currents in the heterologous expression experiments.

It is conceivable that the macrodeletions with the loss of a large stretch of peptides would have impact on protein structure and serious functional effect. By contrast, the fact that the microdeletion and single deletion mutants also resulted in the complete loss-of-function is more striking, especially for the two single deletions (p.M400del and p.I1772del) locating at transmembrane domain DIV/S6. Analogous to p.I1772del, an in-frame deletion in the identical DIV/S6 in Na_v_1.6 (p.I1750del), which is a spontaneous mouse variant, was reported to be associated with a chronic movement disorder with early onset tremor and adult onset dystonia (Jones et al., 2016). The removal of isoleucine in mouse Na_v_1.6 also exhibited no measurable current in the functional studies (Jones et al., 2016). In addition, no measurable current was previously reported in a DS-associated in-frame deletion variant p.F1289del (Thompson et al., 2012), in which phenylalanine in the transmembrane segment DIII/S3 in Na_v_1.1 was removed. In our case, the microdeletion p.S128_F130del with a combined loss of phenylalanine, leucine, and serine in S1 led to complete loss-of-function. These transmembrane residues, including isoleucine, methionine, phenylalanine and leucine are all hydrophobic and critical for the interaction between transmembrane helices and lipid membrane. However, an exceptional case was found in an in-frame deletion of leucine (p.L955del) within DII/S6 of Na_v_1.7, which showed a gain-of-function with a robust hyperpolarizing shift of activation and slow inactivation (Yang et al., 2013). More efforts are needed to understand the functional architecture of voltage-gated sodium channels.

The variant p.T303_R322del is expected to shorten the membrane-reentrant P-loop rather than affect transmembrane helices. The P-loop is the linker between S5 and S6, forming a selectivity filter—a narrow pathway that determines which ion will pass the pore (Catterall et al., 2007; Yang et al., 2009). Functional loss in the p.T303_R322del mutant indicates the non-redundant role of the P-loop for ion conductance. In support of this postulation, it was shown that a five amino acids in-frame deletion of P-loop in a p.R1370-L1374del of Na_v_1.7, which is associated with channelopathy-associated insensitivity to pain disorder, also resulted in a normally expressed but non-functional channel (Cox et al., 2010). Likewise in Na_v_1.5, a heterozygous in-frame deletion p.N1380del that was associated with cardiac conduction disturbance and ventricular tachycardia exhibited no detectable current (Yang et al., 2017), even though only a single amino acid was removed. Together with previous experimental mutagenesis and clinical studies (Terlau et al., 1991; Favre et al., 1996; Ishii et al., 2017), it has well established that P-loops are critical determinants of catalytic permeation properties of Na^+^ channels, but their precise structure-function and deletion-phenotype relationships remain largely unknown.

The most puzzling result in the study is that the truncating mutant (p.M1619Ifs*7), that is expected to delete the S4–S6 pore-forming segments in DIV and the whole C-terminal tail, retained partial channel function. According to several expression and functional studies in the truncated Na_v_, most of the truncation variants would fail to produce functional channel (e.g., hNa_v_1.1-p.R1234*), except those occurring at the C-terminal tail (e.g., hNa_v_1.1-p.R1892*, hNa_v_1.5-p.R1860Gfs*12) (Sugawara et al., 2003; Bechi et al., 2012; Brunklaus et al., 2020). However, the Na^+^ current in the p.M1619Ifs*7 transfected cell was observed in this study, although the current remarkably decreased to 24% of the WT. The evidence proved that the truncated channel without last pore-forming segments still retained the basic biophysics of Na_v_1.1. A possible explanation could be that new assembly of the remaining segments is capable of forming a functional channel.

Several mechanisms might underlie the non-functional Na_v_1.1 channel. First, as Na_v_ channel opening is determined by a series of gating checkpoints in the transmembrane and cytosolic regions, disruptions caused by missense or deletion variants at these gating checkpoints would definitely impact on the functioning of Na_v_1.1 channel. For example, the residues in the pore regions are highly conservative and determine ion selectivity and permeation, and the voltage-sensing domains determine the right response to membrane potential. Secondly, integrity of the functional motifs that determine mRNA expression, splicing, and protein trafficking has been destroyed. Thirdly, a local misfolding of residues disturbs the topology of the adjoining functional motifs and misleads gating motions. In our study, p.T303_R322del, p.M400del, p.V1335_V1428del, and p.I1772del affect the integrity of the pore-forming regions. This is consistent with the roughly loss-of-function changes found in missense variants in these gate checkpoints (Ohmori et al., 2006). However, both p.S128_F130del and p.T160_Y202del transformed the α helices into flexible loops according to the structural modeling (Figure 4), which would have impacts on overall arrangements of different segments. As functional motifs of a protein are usually a fixed order of the residues, deletions could be at higher risk of disrupting the order, thereby causing more deleterious effects on function or structure, when compared to missense or truncation mutations.

It has been suggested that changes in amino acid sequence have variable functional effects on sodium channels, with a mixture of loss-of-function and gain-of-function effects (Yamakawa, 2006; Gataullina and Dulac, 2017). The SMEI-associated *SCN1A* variants seem to be more closely correlated with haploinsufficiency for Na_v_1.1, which caused by deleterious nonsense and frameshift variants in *SCN1A* (Gambardella and Marini, 2009; de Jonghe, 2011), and missense variants exhibiting remarkably attenuated or barely detectable sodium currents (Sugawara et al., 2003). Studies on heterozygous *SCN1A*± mice demonstrated that substantially reduced Na^+^ current density with a loss of sustained high-frequency firing of action potentials were found in hippocampal and cortical interneurons, possibly responsible for the spontaneous seizure phenotypes in the *SCN1A*± mice (Yu et al., 2006). In agreement with the previous result, the six in-frame deletion mutants in this study were non-functional and mostly associated with SMEI, except that two variants, p.T160_Y202del and p.I1772del, which affect pore regions, were associated with FS+ and PEFS+, respectively. The variable phenotypes might be explained by the role of genetic modifiers and molecular interactions. It remains a challenging conundrum for researchers about how the common loss-of-function in Na_v_1.1 results in different phenotypes.

This study provides some new insights into the effect of in-frame deletion on the biological functions of Na_v_1.1, suggesting a complete loss-of-function of channel and their associations with clinical phenotypes including SMEI, FS+, and PEFS+. However, we only characterized the functional properties in the six out of 44 in-frame deletions identified to date. With more deletion variants identified and functionally characterized, the relationship among deletion pattern, functional changes, and clinical phenotypes would become more clear.

## Data Availability Statement

The original contributions presented in the study are included in the article/supplementary material, further inquiries can be directed to the corresponding author/s.

## Ethics Statement

The studies involving human participants were reviewed and approved by Ethics Committee of The Second Affiliated Hospital of Guangzhou Medical University. Written informed consent to participate in this study was provided by the participants’ legal guardian/next of kin.

## Author Contributions

TS and NH contributed to the conceptualization and funding acquisition. TS and J-YW designed the study, while the other authors performed the experiments and/or analyzed the data, and contributed to the manuscript draft. All authors contributed to the article and approved the submitted version.

## Conflict of Interest

The authors declare that the research was conducted in the absence of any commercial or financial relationships that could be construed as a potential conflict of interest.

## Publisher’s Note

All claims expressed in this article are solely those of the authors and do not necessarily represent those of their affiliated organizations, or those of the publisher, the editors and the reviewers. Any product that may be evaluated in this article, or claim that may be made by its manufacturer, is not guaranteed or endorsed by the publisher.

## Footnotes

1
http://scn1a.caae.org.cn/

2
http://www.ncbi.nlm.nih.gov/

3
https://zhanglab.ccmb.med.umich.edu/I-TASSER/
